# Chronic contained rupture of abdominal aortic aneurysm that developed from chronic abdominal aortic dissection

**DOI:** 10.1016/j.jvscit.2021.01.007

**Published:** 2021-02-10

**Authors:** Shogo Oyama, Shingo Ohuchi, Takeshi Arai, Yukinobu Ito

**Affiliations:** a Department of Cardiovascular Surgery, Nakadori General Hospital, Akita, Japan; b Department of Cellular and Organ Pathology, Akita University Graduate School of Medicine, Akita, Japan

**Keywords:** Abdominal aneurysm, Chronic contained rupture, Chronic aortic dissection

## Abstract

A chronic contained rupture is an extremely rare subtype of abdominal aortic aneurysm rupture. We report the case of a 59-year-old man with a medical history of traumatic lumber fracture 7 years ago. He presented to us with an asymptomatic irregular abdominal aortic aneurysm, and surgery was performed 1 week after he was hospitalized. Based on the medical history, imaging, blood tests, and pathologic results, we determined that the chronic contained rupture progressed from a localized abdominal aortic dissection. This case illustrates the need to follow the morphology of aortic aneurysms if chronic abdominal aortic dissection is observed.

Chronic contained rupture (CCR) is an extremely rare subtype of abdominal aortic aneurysm (AAA) rupture. Jones et al^1^ cited the existence of a known AAA as a criterion for the identification of CCR. This report discusses a case of CCR that developed from a chronic abdominal aortic dissection. The patient provided informed consent for the publication of his case details and images.

## Case report

**Fig 1:**
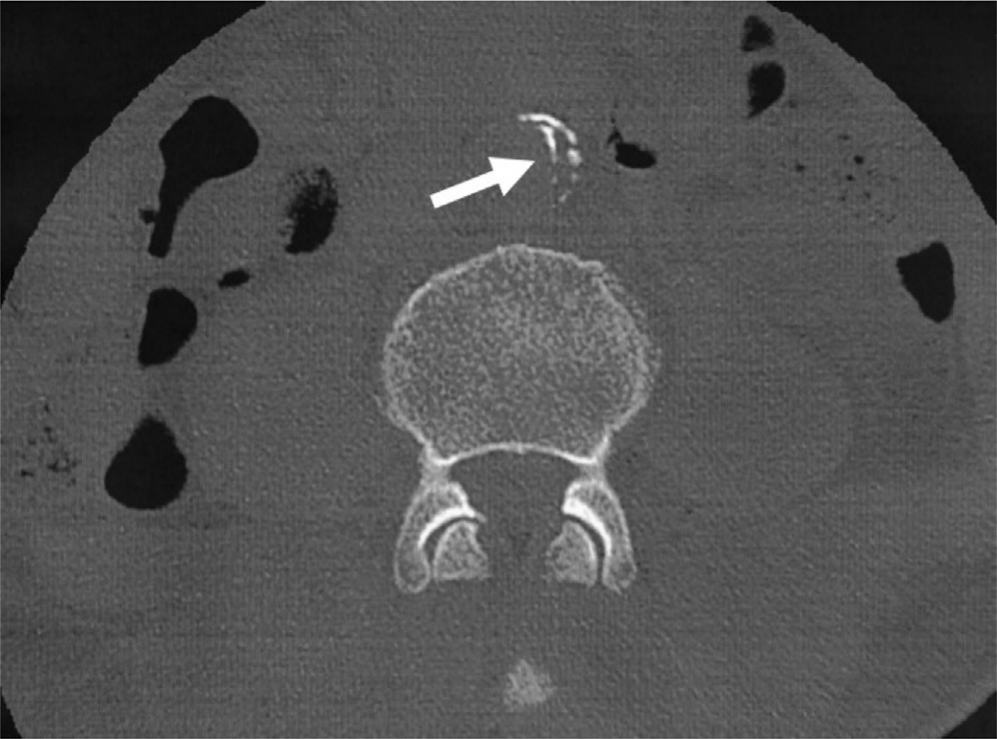
Inward displacement of intimal calcification (⇨) as indicated on the computed tomography (CT) scan performed by the patient's previous doctor. Findings suggested local abdominal aortic dissection.

We report the case of a 59-year-old man with hypertension and dyslipidemia, both of which were being treated by his primary physician. The patient suffered a traumatic lumbar fracture in 2013 owing to a fall and underwent surgery. Computed tomography (CT) scans performed at the time were focused on the lumbar spine, so other organs were barely included in the image range and no contrast medium was used. However, when carefully observed retrospectively, the CT scan showed a partial inward deviation of the intimal calcification of the abdominal aorta, suggesting a localized dissection of the abdominal aorta (Fig 1). However, there was no mention of localized aortic dissection, because the dissection was fairly localized and did not show any abnormal findings, such as aneurysm formation. In addition, the adventitia causing the aortic dissection was also calcified. The findings suggested a chronic aortic dissection that occurred even before the trauma, rather than an acute aortic dissection that occurred at the time of the trauma.

In March 2020, the patient was admitted to the emergency department of a nearby general hospital for vomiting. He had no abdominal pain and was subsequently diagnosed with acute gastroenteritis and prescribed oral medication. On physical examination, palpation of the abdomen revealed a pulsating mass, and the patient subsequently consulted his family doctor. After abdominal ultrasound examination, the patient's doctor diagnosed him with an AAA. He subsequently referred the patient to another hospital for a contrast CT scan and a full workup of the AAA. After the enhanced CT scan, the patient was referred to our hospital, where we determined that urgent treatment was necessary owing to the irregular nature of the aneurysm.

Although he did not have any findings suggestive of an infectious disease such as fever or pain, we first considered the possibility of an infectious aortic aneurysm because it was an irregular aneurysm. Upon examination, a pulsating abdominal mass was found, but without tenderness at the site. We conducted a screening test to locate the source of infection. Furthermore, a blood culture was performed, and the results were negative. An oral examination was performed considering the possibility that the oral infection had spread to the aorta and become an infectious aortic aneurysm. Multiple cavities and loose teeth were noted. Although not deemed to be a source of infection, the teeth were extracted, and the patient was prescribed an antibacterial to prevent future exacerbation. To rule out other infections, further laboratory work included a complete blood count, renal function, liver function, erythrocyte sedimentation rate, C-reactive protein, and IgG-4, all of which were found to be within normal limits.

**Fig 2:**
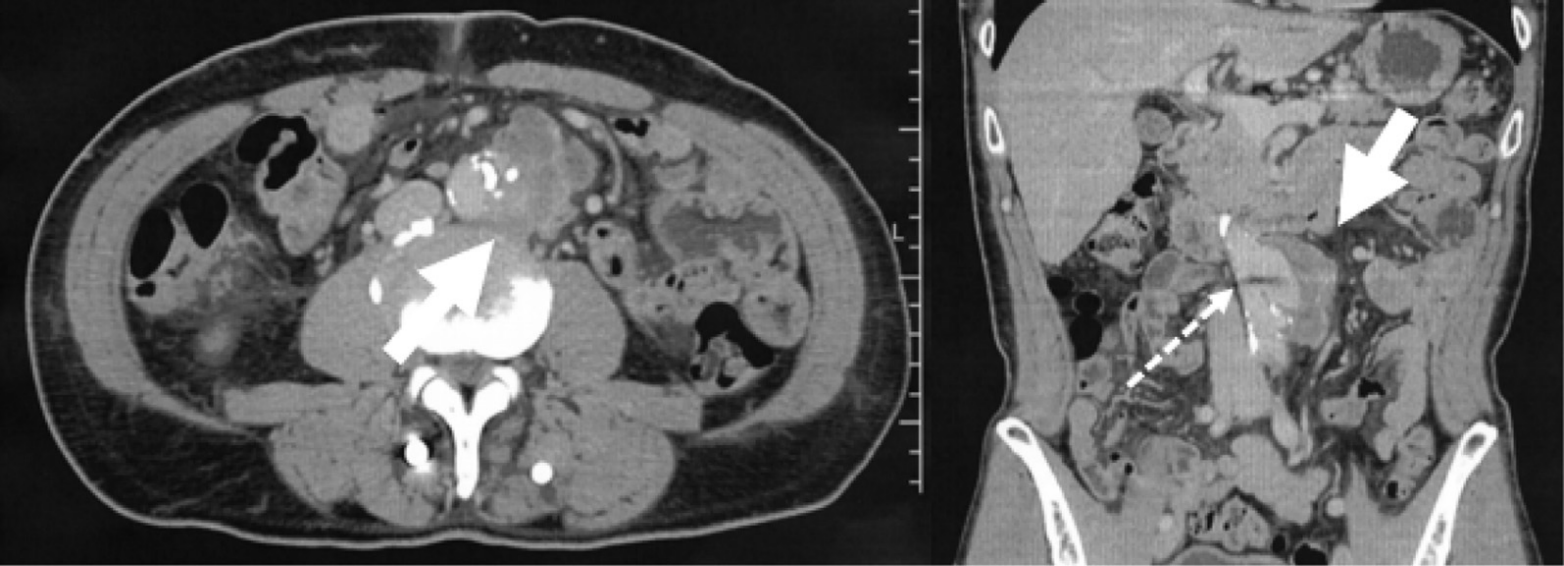
An irregular abdominal aortic aneurysm (AAA) was found in the infrarenal aorta (⇨) suggesting a localized aortic dissection. (⇢) Metal artifacts in the spine.

A CT scan revealed an irregular AAA with a maximum short diameter of 36 mm within the infrarenal aorta (Fig 2). Furthermore, part of the abdominal aortic intima calcifications were displaced inward, suggesting aortic dissection.

A laparotomy was performed by midline abdominal incision. There was no thickening of the arterial wall or retroperitoneal adhesions, as found in inflammatory aneurysms; however, part of the abdominal aneurysm wall was firmly adhered to the surrounding tissue. When the aorta was cross-clamped below the renal artery and the aneurysm was cut open, a partial defect in the intima was observed at the point where CT findings had suggested an aortic dissection. The inside of the false lumen was filled with a dark red thrombus, and a partly organized thrombus was also observed. The aorta was trimmed below the renal artery, and a Y graft replacement (J graft 16 × 8 mm) was performed. The distal side was anastomosed to the common iliac artery on both sides.

**Fig 3:**
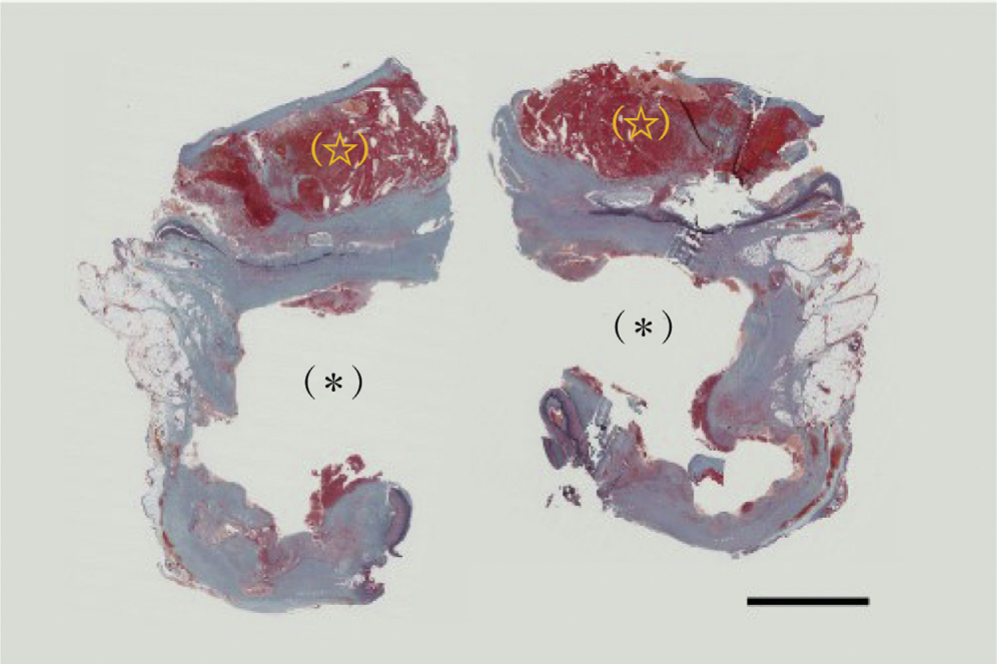
The medial elastic fibers of the aorta were thin. (∗) The lumen developed from a contained rupture. (☆) The lumen was formed of fibrous tissue outside the natural lumen. (Elastica-Masson stain). 5 mm.

Pathologic examination revealed that the medial elastic fibers of the aorta were thin. A vascular lumen was formed by fibrous tissue outside the medial elastic fiber of the aorta. Infiltrating inflammatory cells and macrophages that phagocytosed hemosiderin were found in the surrounding area and wall structure. No elastic fibers were found on the wall of the vessel lumen (Fig 3). From these observations, it was judged that the vascular cavity was formed owing to the collapse of the wall structure of the aortic aneurysm.

No bacteria were detected in the thrombus culture obtained during surgery. One week after surgery, a CT scan confirmed that there were no further complications, and the patient was discharged 8 days postoperatively.

## Discussion

Differentiation of an irregular aneurysm, such as in this case, may be due to CCR or a sealed rupture, an infectious aortic aneurysm like syphilis, an inflammatory aneurysm, or a malignant tumor such as an angiosarcoma.^1^, ^2^, ^3^, ^4^ Differentiation based on imaging findings can be difficult, and comprehensive judgment is required through the use of screening markers such as blood cultures, C-reactive protein, erythrocyte sedimentation rate, and IgG4. This case was diagnosed as CCR, because both the blood culture and inflammatory findings were negative on preoperative examination. There was no infection, inflammation, or tumor present intraoperatively, and disrupted elastic fibers and collapse of the aortic wall structure were confirmed histopathologically.

AAA has been cited as one of the criteria required for the diagnosis of CCR. This case had never been identified as an AAA in the past. Therefore, we focused on the patient's medical history that included a traumatic lumbar fracture. Therefore, we decided to carefully examine the presence of abnormal structural changes in the abdominal aorta that had not been previously noted on a CT scan performed during the surgical treatment of lumbar rupture. We were then able to detect localized aortic dissection in the infrarenal abdominal aorta on that CT scan. This location was considered to coincide with the location where the localized abdominal aortic dissection could be detected by a CT scan performed for a detailed examination of the AAA. However, the adventitia of the abdominal aortic localized dissection was calcified, suggesting a chronic etiology. Therefore, we considered traumatic localized dissection, but it was thought that a chronic, asymptomatic localized dissection was accidentally photographed by the CT scan performed at the time of trauma.

From these observations, we considered that this irregularly shaped AAA was formed from a chronic localized abdominal aortic dissection. Intraoperative findings showed a defect in the intima at the same site, which seemed to be a rupture from a penetrating atherosclerotic ulcer.^5^ However, pathologic examination revealed that the medial elastic fibers were thin and disrupted and, thus, deemed to be a tear in the aortic dissection. The wall of the saccular aneurysm and the irregular mass seen on the CT scan were found to have no elastic fibers on the pathologic examination. As such, the AAA rupture was deemed to be a CCR owing to the collapse of the aortic wall structure that developed asymptomatically from a chronic abdominal aortic dissection.

## Conclusions

We encountered a case of asymptomatic CCR that developed from a chronic abdominal aortic dissection. If chronic abdominal aortic dissection is observed, it is necessary to carefully follow the morphology of the aortic aneurysm.
